# Pregnancy-Related Cardiac Adaptation and Postpartum Echocardiographic Findings in Repaired Tetralogy of Fallot: A Study Integrated with ESC 2025 Recommendations

**DOI:** 10.3390/medicina62030437

**Published:** 2026-02-26

**Authors:** Fatma İşlek Uzay, Mete Sucu, Aslı Sena Alagöz, Süleyman Cansun Demir, İsmail Cüneyt Evrüke, Emre Yalçın, Özge Keleş Bayer

**Affiliations:** Department of Obstetrics and Gynecology, Division of Perinatology, Cukurova University School of Medicine, 01330 Adana, Türkiye; metesucu@yahoo.com (M.S.); asliclkrrr@gmail.com (A.S.A.); cansundemir@gmail.com (S.C.D.); cuneytevruke@gmail.com (İ.C.E.); dremreyalcin23@gmail.com (E.Y.); ozgekeles89@gmail.com (Ö.K.B.)

**Keywords:** tetralogy of fallot, surgically repaired, pregnancy outcome, postpartum echocardiography, patient care team

## Abstract

*Background and Objectives*: To evaluate pregnancy outcomes and transthoracic echocardiographic (TTE) findings during the antenatal and postpartum periods in women with repaired Tetralogy of Fallot (ToF) who delivered at Çukurova University Faculty of Medicine, Balcalı Hospital, between 2011 and 2025 and to interpret these findings in the context of the 2025 European Society of Cardiology (ESC) recommendations. *Materials and Methods*: This single-center retrospective cohort study undertaken between 2011 and 2025 included 11 pregnant women with surgically repaired ToF. Maternal demographic characteristics, obstetric outcomes, mode of delivery, neonatal outcomes, and antenatal TTE parameters were recorded. Cardiac measurements obtained during pregnancy were compared with postpartum TTE findings performed 3–6 months after delivery to assess pregnancy-related cardiac adaptation and recovery. *Results*: A total of 11 pregnancies in women with repaired ToF were analyzed. Nine pregnancies resulted in live births, while one ended in missed abortion at 9 + 2 weeks and one in intrauterine fetal demise at 34 + 2 weeks. Among live births, the mean gestational age was 36 + 2 weeks and the mean birthweight was 2865 g, with a preterm delivery rate of 55.6%. Cesarean section was performed in 70% of cases, while 30% delivered vaginally. During pregnancy, the mean left ventricular ejection fraction was 62.6%, and residual tricuspid regurgitation was the most frequently observed echocardiographic finding. Postpartum TTE evaluations indicated that echocardiographic parameters were largely stable over the observation period, with no numerical change and no clear evidence of deterioration in ventricular function or progression of valvular regurgitation. *Conclusions*: Despite successful surgical repair, pregnancy may still pose potential risks for women with ToF, underscoring the importance of individualized, multidisciplinary management. In this cohort, pregnancy appeared to be generally well-tolerated when care was provided in accordance with contemporary ESC recommendations. The observation of preserved ejection fraction and overall stable right ventricular function in the early postpartum period suggests that favorable maternal cardiac adaptation may be achievable in carefully selected patients. Early postpartum echocardiographic assessment may be useful for identifying functional changes and informing structured long-term follow-up strategies.

## 1. Introduction

Tetralogy of Fallot (ToF) is the most common cyanotic congenital heart disease. Structurally, it involves four pathologies: ventricular septal defect, right ventricular outflow tract obstruction, overriding aorta, and right ventricular hypertrophy. Advances in pediatric cardiac surgery and perioperative care have significantly improved long-term survival in patients with ToF, allowing women, in particular, to reach childbearing age.

Long-term complications are generally related to functional failure of the right ventricular (RV) outflow tract and its secondary effects on ventricular myocardial function [1,2].

Pregnancy brings with it many changes in the circulatory system: increased blood volume, increased load on cardiac output, changes in vascular tone and physiological changes due to hormonal effects [3]. Pregnancy is a process that causes significant hemodynamic changes even in normal physiology; changes such as a 40–50% increase in blood volume, an increase in cardiac output and a decrease in pulmonary vascular resistance place additional burden on the cardiovascular system in women with congenital heart disease [3,4]. Therefore, close monitoring of right ventricular function, residual valve pathologies, and pulmonary hypertension is necessary throughout pregnancy and postpartum in pregnant women with operated ToF. In women with ToF, these changes may interact with existing cardiac conditions such as residual lesions (e.g., pulmonary regurgitation), right ventricular dysfunction, and arrhythmia predisposition [5,6]. In women with congenital heart disease—especially in cases of ToF with residual lesions after surgical repair—pre-pregnancy counseling, multidisciplinary follow-up, and individualized risk assessment based on cardiac function and the presence of residual defects are of great importance [4,7].

The postpartum period represents a particularly vulnerable phase, as the rapid reversal of pregnancy-related hemodynamic changes may unmask subclinical ventricular dysfunction or exacerbate residual lesions. Despite this, postpartum cardiac evaluation is not consistently performed or reported, resulting in a significant gap in understanding early post-pregnancy cardiac adaptation in women with repaired ToF.

Although women with surgically repaired ToF generally tolerate pregnancy, this group exhibits a heterogeneous residual anatomic structure, and pregnancy-related risks are not entirely predictable. Residual lesions such as right ventricular volume overload, pulmonary insufficiency, tricuspid regurgitation, and right heart dilatation may become evident with hemodynamic stress during pregnancy and may impact ventricular function in the postpartum period. However, data evaluating the long-term, and particularly postpartum, effects of pregnancy on cardiac function in these patients are quite limited. Studies reported in the literature indicate that most women with repaired ToF tolerate pregnancy relatively well, with a low major maternal mortality rate; however, the risk of fetal/neonatal adverse outcomes, such as fetal loss, prematurity, low birth weight, and congenital heart anomalies, may be increased [8]. These adverse outcomes include spontaneous abortion, premature birth, fetal death, neonatal death, and the presence of congenital heart anomalies [9,10].

Hemodynamic changes increase cardiac output, and changes in vascular resistance that occur during pregnancy place additional stress on the cardiovascular system, which can affect both maternal and fetal health [5]. In fact, it has been reported that up to 8% of patients with ToF will experience cardiac complications during pregnancy [11].

Pulmonary insufficiency or stenosis can lead to progressive RV dysfunction and failure, progressive tricuspid valve insufficiency, arrhythmias, and sudden death [12,13].

Pulmonary regurgitation and right ventricular dysfunction, in particular, are considered important predictors of increased risk of fetal and maternal complications. Furthermore, it is noteworthy that pregnancy carries a higher risk in women with unrepaired ToF and that pregnancy after surgical repair is the primary goal in this group [9,14].

Increased right ventricular pressure and volume load following pulmonary valve regurgitation leads to long-term right ventricular remodeling. Although some clinicians recommend pulmonary valve replacement (PVR) before pregnancy, data evaluating the impact of this practice on pregnancy outcomes are limited [13].

In our study, we aimed to examine the pregnancy outcomes of patients diagnosed with operated ToF along with their neonatal outcomes. By comparing the pregnancy outcomes with the recommendations of the ESC 2025 guidelines, we aimed to contribute to this patient group, which has limited data in the literature [15]. The ESC 2025 Pregnancy and Cardiovascular Diseases Guidelines recommend a multidisciplinary “Maternal Cardiac Team” approach for pregnant women with congenital heart disease, emphasizing the importance of risk assessment and individualized management [15]. However, the guideline falls short of providing specific, high-level evidence recommendations for operated ToF; it specifically highlights the lack of data on changes in right ventricular function in the postpartum period. This complicates clinicians’ decision-making regarding both pregnancy management and postpartum follow-up.

According to the recommendations of the ESC 2025 guide, all women with congenital heart disease (CHD) who wish to conceive should receive counseling from a maternal cardiac team. This multidisciplinary team is clearly emphasized in the guidelines. Vaginal birth is recommended for most women with CHD. Therefore, cesarean delivery is not always mandatory; vaginal delivery may be preferred if there are no obstetric reasons [15].

In this study, maternal, fetal, and neonatal pregnancy outcomes were evaluated in accordance with the ESC 2025 recommendations in women with Tetralogy of Fallot surgically repaired in childhood, and postpartum cardiac function changes during pregnancy were systematically examined [15].

However, the general summary of the ESC 2025 Guidelines provides specific, high-level opportunities to address Tetralogy of Fallot with repaired circulation, extended cardiac adaptation, and postnatal outcomes. This can largely be attributed to the short patient durations, the heterogeneity of surgical techniques and remaining lesions, and the lack of regular systematic postnatal cardiac assessment in published series. Consequently, clinical decision-making in this subgroup is often based on cardiac inferences from broader congenital disease categories rather than on case-specific data [15].

The aim was to shed light on the reproductive prognosis of this unique and growing patient group by combining clinical findings with the existing literature data. The existing literature on pregnancy outcomes in operated ToF cases is limited due to both the relatively small number of patients and significant variability in approaches across centers. Furthermore, retrospective and heterogeneous data structures hinder the development of clear recommendations specific to this subgroup.

Another unique aspect of our study is the comparison of the findings with the recommendations in the ESC 2025 guidelines and the demonstration of the clinical value of postpartum echocardiography in this patient group [15].

## 2. Materials and Methods

This retrospective study was conducted among pregnant women who presented to the Perinatology Department of the Department of Obstetrics and Gynecology at Çukurova University Faculty of Medicine between 1 January 2011 and 31 December 2025.

Ethical approval for this study was obtained from the Çukurova University, Faculty of Medicine Non-Interventional Clinical Research Ethics Committee. The study protocol was reviewed and approved at committee meeting number 162, with decision number 35. Although the study was retrospective in nature and based on the review of existing medical records, ethical approval was sought and obtained to ensure compliance with institutional and international ethical standards. All procedures were conducted in accordance with the principles of the Declaration of Helsinki. Patient data were anonymized prior to analysis, and no intervention beyond standard clinical care was performed.

Patient demographic data, obstetrical course, fetal outcomes, and TTE findings from the pregnancy and postpartum periods were obtained from the electronic patient record system. Inclusion criteria for the study group were a diagnosis of surgically repaired ToF, pregnancy follow-up at our hospital, delivery or termination of pregnancy, availability of TTE data for the year of pregnancy and having a TTE record performed within 3–6 months postpartum. Exclusion criteria for the study were: unrepaired ToF; the presence of a disease in the mother that would affect the growth and development of the fetus such as diabetes, thyroid disease, pre-eclampsia, chronic hypertension, multiple pregnancy, anemia in the fetus, widespread bleeding or infarction areas in the placenta suggesting uteroplacental insufficiency; and the pregnancy being monitored in another center.

For each patient, age, pregnancy history, gestational age, mode of delivery, neonatal birth weight, 1 and 5 min APGAR scores, obstetric complications, presence of intrauterine fetal death or missed abortion, cardiovascular symptoms, and need for maternal–fetal intensive care were recorded (Table 1). Echocardiographic data from pre- and post-pregnancy periods were used to assess ejection fraction, right ventricular size and function, degree of tricuspid regurgitation (TR), degree of pulmonary regurgitation (PR), presence of pulmonary hypertension, and presence of residual lesions. End-diastolic diameter values measured by TTE for right ventricular function were added to Table 2 and Table 3. Data were analyzed using descriptive statistics and presented as percentages and mean ± standard deviation.

Ultrasonographic examinations were performed by perinatology faculty members and physicians in subspecialty residency training. Ultrasonographic examinations were performed either with a GE Voluson 730 Pro ultrasonography device and an RAB 4–8 MHz volumetric convex probe or with a GE E6 ultrasonography device and an RAB 4–8 MHz volumetric convex probe. Images obtained during the examinations are stored in the ViewPoint^®^ 6.0 hospital data processing system.

The study protocol was approved by the Çukurova University Faculty of Medicine Non-Interventional Clinical Research Ethics Committee.

Data were summarized using descriptive statistics. Continuous variables were presented as mean ± standard deviation or median (IQR), and categorical variables were presented as frequencies and percentages. Paired analysis was used to compare echocardiographic parameters during pregnancy and postpartum. Due to limited sample size, advanced statistical modeling was not performed. All analyses were performed using the SPSS 25.0 software package.

## 3. Results

A total of 11 pregnant women with surgically repaired ToF were included in the study. Pregnancy outcomes were evaluated as follows: nine cases had live birth, one case had missed abortion at 9 + 2 weeks, and one case had intrauterine fetal death (IUFD) at 34 + 2 weeks. In cases with live birth, the mean maternal age was 28.9 ± 4.2 years. In the nine liveborn babies, the mean gestational age was 36 + 2 weeks (range: 31 + 2–39 + 1). Mean birth weight was 2865 ± 770 g. Five out of nine were preterm births (<37 weeks), two early preterm (31 + 2; 33 + 0) and three late preterm (35 + 3; 36 + 3; 36 + 4), and four out of nine were term births (≥37 weeks) (44.4%). The missed abortion case ended at 9 + 2 weeks and was not included in the obstetric analyses. Cesarean section was performed for 7/10 (70%), and vaginal delivery was performed for 3/10 (30%). In cases of missed abortion, dilatation and curettage were performed (Table 1).

Indications for cesarean section included the presence of previous surgical repair, obstetric preferences, and fetal indications.

In the nine neonates born alive, APGAR was 7–9 at minute 1 and APGAR at minute 5 was 8–10; the need for neonatal intensive care was limited and no severe neonatal depression was observed.

Pregnancy TTE findings were available for 10 of the 11 patients. Mean ejection fraction (EF) was 62.6% ± 3.7% (range: 58–70%). The most common cardiac findings during pregnancy were: moderate tricuspid regurgitation (TR), mild to moderate pulmonary regurgitation (MPR), mild mitral regurgitation (MR), pulmonary prosthetic valve findings, right ventricular dilatation, and residual structural anomalies (wide VSD patch, ASD sequelae, and overriding aorta).

Pregnancy TTE findings of the IUFD case: pulmonary prosthetic valve, moderate TR, moderate pulmonary hypertension, and mild MR (Table 2).

Postpartum TTE reports of all patients were reviewed (Table 3).

Ventricular function: mean postpartum EF 63% (58–68), with no numerical change observed compared with the gestational period. Right ventricular function was preserved in all patients.

Valve pathologies: moderate TR: five cases, mild TR: three cases, moderate PR: two cases, mild PR: three cases, and mild–moderate MR: five cases. Postpartum TTE evaluations indicated that echocardiographic parameters were largely stable over the observation period with no numerical change and no clear evidence of deterioration in ventricular function or progression of valvular regurgitation.

Mild right heart dilatation persisted in some cases.

Surgery sequelae (VSD patches, ASD sequelae, and overriding aorta) remained stable postpartum.

Postpartum clinical outcomes revealed no major cardiac complications; there were no arrhythmias, acute decompensation or cardiac hospitalizations. When antenatal and postpartum echocardiographic measurements were compared descriptively, changes in key parameters appeared small. Mean ejection fraction showed only limited numerical variation, and absolute changes in right ventricular functional indices and valvular regurgitation grades appeared minimal over the observation period.

In our study, the live birth rate was 81.8%, the preterm birth rate was 55.6%, and the cesarean section rate was 70%. Postpartum ejection fraction appeared preserved and no numerical worsening of valvular pathology was observed. These findings support the clinical suitability of the “pre-pregnancy risk assessment + multidisciplinary follow-up + postpartum cardiac monitoring” approach proposed by ESC 2025 [15].

## 4. Discussion

This study is one of the few to systematically evaluate maternal, obstetric, and postpartum cardiac outcomes in pregnant women diagnosed with surgically repaired ToF.

Previous studies have shown that women with operated ToF generally have a good maternal prognosis with low rates of maternal mortality and major cardiac events. Although the number of patients is limited, our findings, in line with the existing literature, suggest that pregnancy may be generally well-tolerated in many patients within this group, with ventricular function appearing preserved in the postpartum period and numerically no clear indication of progression in residual valvular pathology on descriptive assessment [2,6].

However, fetal and neonatal outcomes in cases with unrepaired ToF are more adverse compared to the general population. A retrospective study by Wang et al. examined 31 pregnancies and reported that rates of prematurity (22.6%), small-for-gestational-age infants (22.6%), and fetal or neonatal death (9.6%) were higher, particularly in unrepaired ToF cases. While no maternal mortality was found, right ventricular outflow tract stenosis, pulmonary regurgitation, and cyanosis were emphasized as primary determinants of adverse fetal outcomes [9].

In a 2021 study evaluating the prediction of maternal cardiac complications, ventricular dysfunction, low saturation, and advanced residual lesions were listed, and the high live-birth rate in our study was associated with the preservation of EF values [16].

In our cohort, the live birth rate was 81.8%, which is comparable to the 85–90% rates reported in larger series including 31 patients [9]. Our preterm birth rate (55.6%) was higher than the 25–45% range reported in many studies; however, it has previously been shown that postoperative pulmonary regurgitation, right ventricular volume overload, and the hemodynamic stress of pregnancy increase susceptibility to preterm delivery. In this context, features such as moderate tricuspid regurgitation and pulmonary hypertension in our cases influenced the decision for cesarean delivery based on cardiology consultation. Notably, in women with repaired ToF who have residual right heart loading, obstetric complications such as higher rates of preterm birth may occur [5,6].

Although the presence of an IUFD case is rare, fetal loss rates in pregnancies with ToF have been reported in the literature to range between 2% and 4% [17,18]. Cases complicated by pulmonary hypertension and advanced tricuspid regurgitation appear to be more prone to fetal complications, consistent with previous reports. In our series, the presence of moderate pulmonary hypertension and valvular abnormalities in the IUFD case supports this observation [6,10].

In our study, the cesarean section rate was 70%, which appears higher than the rates generally reported in the literature (40–55%) [18]. However, the ESC 2025 guidelines recommend vaginal delivery in women with repaired ToF unless there are clear obstetric or cardiac indications. It is nevertheless emphasized that clinician preference, prior obstetric history, and fetal indications may contribute to higher cesarean rates [15].

The volume load imposed on the right ventricle (RV) during pregnancy represents the most critical physiological stressor for women with repaired ToF [19]. Previous studies have shown that pregnancy may cause mild and transient changes in RV strain parameters, which generally return to normal in the postpartum period [20]. In our findings, the absence of clear deterioration in right ventricular function during pregnancy and the observation of preserved ejection fraction are consistent with these reports.

The most common residual lesion observed on pregnancy echocardiograms was moderate tricuspid regurgitation [2,5]. This finding represents a typical pattern reflecting right ventricular volume loading in repaired ToF and is consistent with the literature. In the few cases with a pulmonary prosthetic valve, valvular function remained stable during pregnancy.

The unique aspect of our study is that all patients underwent echocardiographic evaluation in the postpartum period (3–6 months). In the literature, data on postpartum cardiac function in pregnant women with repaired ToF are quite limited and most studies do not assess the early post-pregnancy period. In our cohort, the mean postpartum ejection fraction appeared preserved (63%), right ventricular function remained numerically stable, and no clear increase was observed in the degree of tricuspid, pulmonary, or mitral regurgitation. No new-onset valvular pathology or new findings related to surgical sequelae were identified. No major postpartum cardiac complications occurred. These findings support the ESC 2025 guideline recommendation for postpartum evaluation and demonstrate that such assessment serves as both a reassuring measure and a clinically informative tool in women with repaired ToF [4]. Risk stratification and pre-pregnancy assessment emphasize collaboration between cardiology and obstetrics and incorporate ventricular function, arrhythmias, and hemodynamic status into decision-making. Postpartum follow-up and long-term risk assessment are essential to minimize adverse pregnancy outcomes, and cardiovascular risk evaluation should be performed in these patients [7,15].

This risk is particularly pronounced in patients who have residual pulmonary regurgitation, right ventricular outflow tract obstruction, or ventricular dysfunction following surgical repair [5,6]. Therefore, in women with ToF who are planning pregnancy, preconception risk assessment, detailed cardiac functional evaluation, multidisciplinary follow-up, and coordinated patient care are of critical importance [7,15,20]. Although the ESC 2025 recommendations provide important guidance under the broader category of congenital heart disease, specific evidence regarding the management of pregnant women with repaired ToF remains limited. By presenting the pregnancy outcomes of this unique subgroup, our study contributes valuable insight to both clinical decision-making processes and guideline implementation [15].

Considering all these findings, our study demonstrates that pregnancy outcomes in women with repaired ToF are generally favorable, although rates of preterm birth and cesarean delivery may remain high. This underscores the importance of the multidisciplinary approach emphasized in the ESC 2025 guidelines and further supports the need for comprehensive preconception cardiac evaluation [2,15].

Our study has several inherent limitations. First, the small sample size limits the statistical power and generalizability of the findings. Second, selection bias is likely present, as only patients with complete postpartum data were included. Complicated cases might have been lost to follow-up. Third, the absence of a control group prevents definitive comparisons regarding the specific impact of pregnancy versus the natural course of the disease. Finally, as with all retrospective imaging studies, inter-observer variability in echocardiographic measurements cannot be entirely excluded.

Although our results suggest that pregnancy is well-tolerated in this specific cohort, these findings should be interpreted with caution due to the limited sample size. Clinicians should continue to provide comprehensive pre-conception counseling and maintain strict multidisciplinary surveillance during the postpartum period rather than assuming a universally low-risk profile for all repaired ToF patients.

## 5. Conclusions

Our study provides real-world clinical data on pregnancies in women with repaired ToF, enabling evaluation of how broadly applicable the guideline-based recommendations are to this specific subgroup. The findings we obtained are largely consistent with the ESC 2025 recommendations, particularly regarding preconception assessment, multidisciplinary follow-up, and delivery planning [15]. Pregnancy is generally well-tolerated in women with surgically repaired ToF when adequate follow-up conditions are ensured, and most cases can be completed safely [4]. The high live-birth rate observed in our cohort, the increased frequency of preterm birth consistent with the literature and the absence of major maternal cardiac complications suggest that pregnancy in this patient group may be managed with acceptable outcomes in a carefully controlled clinical setting. In this limited cohort, pregnancy appeared to be well-tolerated.

For women with ToF who are planning pregnancy, preconception risk assessment, cardiac functional analysis, and multidisciplinary follow-up are crucial. Although pregnancy in women with repaired TOF is reported to be well-tolerated under appropriate conditions, pregnancy outcomes can vary depending on individual risk profiles. Therefore, comprehensive evaluation and monitoring by a multidisciplinary team structured in accordance with ESC 2025 recommendations is of great importance. Such an approach can contribute to safer management of pregnancy in women with repaired or unrepaired ToF who have reached reproductive age, taking into account both maternal and fetal well-being [2,6,9,15]. Comparison of antenatal and postpartum echocardiographic findings suggests that ventricular function appears largely preserved in the early postpartum period. Numerically, no clear progression in residual tricuspid or pulmonary insufficiency was observed, and right ventricular function generally appeared stable. These findings are consistent with the ESC 2025 recommendations, which emphasize the importance of prenatal assessment, multidisciplinary management during pregnancy, and structured postpartum cardiac monitoring [15]. In patients with surgically repaired ToF, pregnancy has been observed to have an acceptable course in most cases when appropriate clinical follow-up and careful monitoring are provided. Overall, our study presents real-life observations regarding pregnancy outcomes in this patient group and provides findings supporting the clinical applicability of ESC 2025 recommendations in terms of multidisciplinary assessment, careful monitoring, and delivery planning. However, prospective studies with larger sample sizes are needed to increase the generalizability of these findings and to reach more definitive conclusions [2,6,9].

## Figures and Tables

**Table 1:** Pregnancy and delivery outcomes in women with repaired Tetralogy of Fallot.

Patient	Obstetric History	Gestational Age	Birthweight	APGAR (1–5 min)	Mode of Delivery
1	G1	36 + 4	2635 g	9–10	Cesarean section
2	G6P2L2A3	35 + 3	2420 g	8–9	Cesarean section
3	G2A1	36 + 3	3210 g	9–10	Vaginal delivery
4	G2P1L1	39 + 1	3700 g	9–10	Cesarean section
5	G1	38 + 6	3240 g	8–9	Cesarean section
6	G1	31 + 2	1650 g	6–8	Vaginal delivery
7	G1	37 + 5	4030 g	7–9	Cesarean section
8	G1	33 + 0	1750 g	8–9	Vaginal delivery
9	G1	38 + 0	3150 g	7–10	Cesarean section
10	G5P2L1D1A2	9 + 2	—	—	Dilation & Curettage (missed abortion)
11	G1	34 + 0	3000 g	0–0	Cesarean section (IUFD)

**Table 2:** Antenatal TTE findings of the patients.

Patient No	Pregnancy EF (%)	RV Function	RV Diameter	TR Grade	PR Grade	MR Grade	Additional Structural Findings
1	62	Normal	28 mm	Moderate	Mild	Mild	Residual ASD
2	60	Normal	43 mm	Mild	Mild	None	Overriding aorta
3	58	Normal	25 mm	Moderate	Moderate	Mild	VSD patch
4	63	Normal	42 mm	Moderate	Mild	None	Right heart dilation
5	65	Normal	40 mm	Mild	None	Mild	VSD patch
6	61	Normal	29 mm	Mild	Mild	Mild	Residual ASD
7	70	Normal	41 mm	Moderate	Moderate	Moderate	Right heart dilation
8	64	Normal	42 mm	Moderate	Mild	Mild	VSD patch
9	60	Normal	51 mm	Mild	Mild	None	Overriding aorta
10 (Missed abortion)	59	Normal	29 mm	Mild	None	None	Normal postoperative anatomy
11 (IUFD)	58	Normal	45 mm	Moderate	None	Mild	Pulmonary prosthetic valve, pulmonary hypertension

EF: Ejection fraction, RV: Right ventricle, TR: Tricuspid regurgitation, PR: Pulmonary regurgitation, MR: Mitral regurgitation.

**Table 3:** Postpartum TTE findings of the patients.

Patient No	Postpartum EF (%)	RV Function	RV Diameter	TR Grade	PR Grade	MR Grade	New-Onset Pathology	Clinical Postpartum Cardiac Event
1	63	Normal	30 mm	Moderate	Mild	Mild	None	None
2	62	Normal	40 mm	Mild	Mild	None	None	None
3	60	Normal	26 mm	Moderate	Moderate	Mild	None	None
4	65	Normal	40 mm	Moderate	Mild	None	None	None
5	64	Normal	34 mm	Mild	None	Mild	None	None
6	61	Normal	30 mm	Mild	Mild	Mild	None	None
7	68	Normal	41 mm	Moderate	Moderate	Moderate	None	None
8	63	Normal	44 mm	Moderate	Mild	Mild	None	None
9	62	Normal	45 mm	Mild	Mild	None	None	None
10	60	Normal	31 mm	Mild	None	None	None	None
11	58	Normal	46 mm	Moderate	Mild	Mild	None	None

EF: Ejection fraction, RV: Right ventricle, TR: Tricuspid regurgitation, PR: Pulmonary regurgitation, MR: Mitral regurgitation.

## Data Availability

The data presented in this study are available on request from the corresponding author. The data are not publicly available due to ethical and privacy restrictions related to patient information.
